# The Usefulness of Retinoic Acid Supplementation during In Vitro Oocyte Maturation for the In Vitro Embryo Production of Livestock: A Review

**DOI:** 10.3390/ani9080561

**Published:** 2019-08-15

**Authors:** Sameh A. Abdelnour, Mohamed E. Abd El-Hack, Ayman Abdel-Aziz Swelum, Islam M. Saadeldin, Ahmed E. Noreldin, Asmaa F. Khafaga, Mohsen G. Al-Mutary, Muhammad Arif, El-Sayed O. S. Hussein

**Affiliations:** 1 Animal Production Department, Faculty of Agriculture, Zagazig University, Zagazig 44511, Egypt; 2 Department of Poultry, Faculty of Agriculture, Zagazig University, Zagazig 44511, Egypt; 3 Department of Animal Production, College of Food and Agriculture Sciences, King Saud University, P.O. Box 2460, Riyadh 11451, Saudi Arabia; 4 Department of Theriogenology, Faculty of Veterinary Medicine, Zagazig University, Zagazig 44511, Egypt; 5 Department of Physiology, Faculty of Veterinary Medicine, Zagazig University, Zagazig 44511, Egypt; 6 Department of Histology and Cytology, Faculty of Veterinary Medicine, Damanhour University, Damanhour 22511, Egypt; 7 Department of Pathology, Faculty of Veterinary Medicine, Alexandria University, Edfina 22758, Egypt; 8 Basic Sciences Department, College of Education, Imam Abdulrahman Bin Faisal University, Dammam 31451, Saudi Arabia; 9 Department of Animal Sciences, College of Agriculture, University of Sargodha, Sargodha 40100, Pakistan

**Keywords:** retinoic acid, in vitro, blastocyst production, livestock

## Abstract

**Simple Summary:**

In this review, we provide the previous studies, state-of-the-art practices, and potential implications of retinoic acid for improving in vitro livestock embryo production.

**Abstract:**

Retinoic acid (RA) is an indigenous metabolite and descriptive physiologically functioning constituent of vitamin A. Retinoids were documented as vital regulators for cell development and distinction, embryonic growth, and reproductive function in both male and female livestock. Previously, RA has been shown to have several positive impacts in vivo and in vitro and critically control many reproductive events, such as oocyte development, follicular growth, and early embryonic growth. In addition, RA manages apoptotic signaling and oxidative damages in cells. Recently, RA has been used widely in assisted reproductive technology fields, especially during in vitro embryo development in various mammalian species, including buffaloes, bovine, goats, sheep, pigs, and rabbits. However, the optimum concentration of RA greatly differs based on the condition of maturation media and species. Based on the obtained findings, it was generally accepted that RA enhances nuclear oocyte maturation, cleavage and maturation rates, blastocyst formation, and embryo development. As such, it possesses antioxidant properties against reactive oxygen species (ROS) and an anti-apoptotic effect through enhancing the transcription of some related genes such as superoxide dismutase, prostaglandin synthase, glutathione peroxidase, peroxiredoxins, and heme oxygenase. Therefore, the current review concludes that an addition of RA (up to 50 nM) has the potential to improve the oocyte maturation media of various species of livestock due to its antioxidant activity.

## 1. Introduction 

The in vitro production of embryos (IVP) is a promising approach to assisted reproductive technologies and could lead to genetic improvement in breeding schemes. Current IVP goals are to overcome some of the infertility disorders and generate cloned and/or transgenic animals. However, these processes still have low overall efficiency, which is somewhat due to the incomplete understanding of species-specific demands for in vitro oocyte maturation, fertilization, and embryo culture [1,2,3]. Providing essential nutrients or survival factors to the maturation media has become a broadly applicable approach; this may have the advantages of enhancing in vitro oocyte competence and embryonic growth. In light of this, many approaches have been implemented to explore the impacts of different culture conditions enriched with antioxidants or vitamins on oocyte ripening and embryonic growth in different mammalian species [1,4,5,6,7]. The low efficiency of in vitro embryo production could be attributed to the lack of knowledge on different nutritional requirements for oocyte maturation and embryo development, ROS generation, the difference in time of cytoplasmic and nuclear maturation, the inferior characters of frozen semen, apoptosis, and/or the alteration of transcription gene expression [4,5,7].

Retinoic acid (RA), as a source of vitamin A, plays a pivotal role in reproduction; its presence in excessive or insufficient amounts may result in embryonic loss [5,6,8]. Retinoids collectively contain several features of vitamin A such as retinol; which is the main form of vitamin A and its derivatives (metabolites and analogues). Evidence from numerous reports in different mammalian species concluded that retinoids were involved in in vitro maturation, follicular growth, oocyte development, fertilization, and earlier embryonic growth. In cattle, the level of RA in the ovarian follicular fluid may be considered as an accepted marker for follicular health status, where high estradiol levels are greatly associated with healthy follicles, and the lowest levels indicate atretic follicles or follicle syndromes [9,10,11]. 

In addition, treatment with β-carotene (a precursor to vitamin A found in plants) or RA inhibited fetal reabsorption in rodents [12] improved offspring output in rabbits [13] and enhanced litter size in swine [14]. In ewes, the addition of retinol following natural mating was involved in superovulation and the enhanced competency of morula phase embryos [15]. However, in cattle, RA injection improved the quality of embryos gathered from superovulated individuals [16].

The biosynthesis and excretion of RA in the oviduct and uterus is controlled by the movement of the ovum or embryo inside the female genital tract [17]. Recently, Dyce et al. [18] revealed that RA can improve the meiotic progress, which enhances the differentiation of skin-derived stem cells into oocyte-like cells. This review article provides updates on the methods of retinoic acid supplementation to different oocyte maturation media; investigates the potential ability of retinoic acid to improve the cleavage and maturation rates and blastocyst formation; and illustrates the possible mode of actions, including the improvement of oxidative status, apoptosis, anti-oxidant-related gene expression, and prostaglandin-endoperoxide synthase. 

## 2. Forms of RA 

**Figure 1:**
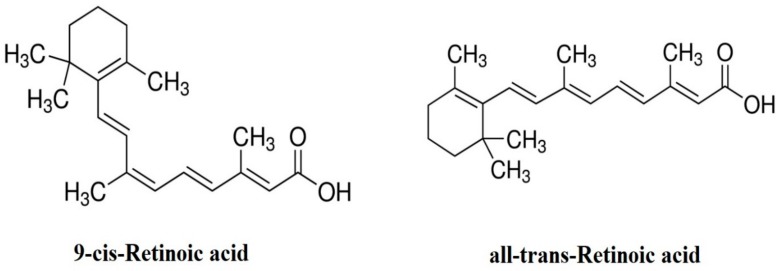
Retinoic acid forms.

**Figure 2:**
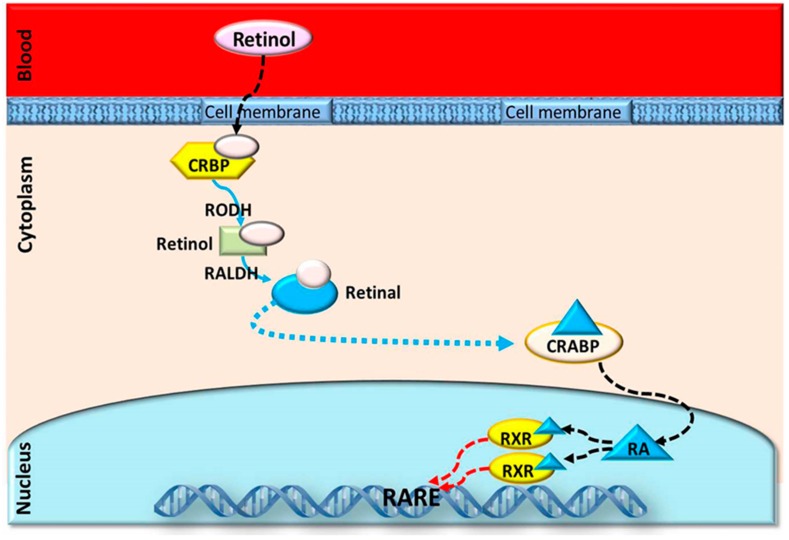
The cellular mechanism of retinoid action. Retinol is taken up from the blood and bound to CRBP (cellular retinol-binding protein) in the cytoplasm. The retinol dehydrogenase (RoDH) enzymes metabolize retinol to retinal, which is then metabolized to RA by the retinaldehyde dehydrogenases (RALDHs). RA is bound in the cytoplasm by cellular RA-binding protein (CRABP). RA enters the nucleus and binds to the RA receptors (RARs) and the retinoid X receptors (RXRs), which themselves heterodimerize and bind to a sequence of DNA known as the RA-response element (RARE). This activates the transcription of the target gene.

Vitamin A is an indispensable nutrient that is well documented for its function in female reproduction, including follicular development, oocyte maturation, steroidogenesis cytoplasmic formation, and embryonic growth. In ruminants, vitamin A is supplemented as β-carotene (from grass), then passed through the intestinal mucosa and enzymatically transformed to retinal, which is converted to retinol [19]. Additionally, RA and β-carotene are contained in the follicular fluid; the internal follicular activity transforms β-carotene into retinol, which can then be transformed into all-trans retinoic acid (AtRA) and 9-cis-retinoic acid (9-cisRA), the active forms of vitamin A (Figure 1) [20]. These active forms might be incorporated into RA receptors (RARs) and retinoid X receptors (RXRs) and combined with the booster areas of some genes to control their expressions [21]. Transcripts of RAR were expressed in bovine cumulus cells, granulosa cells, and oocytes, indicating the biotransformation of vitamin A inside these cells [22,23]. RA was transferred by the blood and then bound to retinol-binding protein (RBP). Cellular retinoic acid-binding proteins and cellular retinol-binding proteins (crbp) have vital roles in intracellular homeostasis, metabolism, and the transport of vitamin A [24]. 9-cisRA and AtRA are intracellular products of RA that control the biological function through their strong interactions with RXRs and RARs, respectively. Ligand-bound RXRs and RARs affect the expression through reacting with the outcome ingredients in the promoter parts of retinoid-modulated genes (Figure 2) [25]. Within the ovarian tissue, both CRBP and RBP are expressed in the granulosa and theca cells and control the movement of retinol from the blood to the growing ovarian follicles [10]. Levels of RBP and its substance retinol in the follicular fluid of large pre-ovulatory bovine ovarian follicles were found to be higher than in the atretic or smaller ovarian follicles [4].

## 3. Effects of RA and Retinol on Oocyte until Blastocyst Formation 

### 3.1. Effects of RA on Oocyte Maturation Rates 

In water buffaloes, Gad et al. [4] found that supplementation with 5 nM of 9-cisRA to the in vitro maturation medium led to a higher oocyte maturation rate. Moreover, treatment with high levels of RA (50 and 200 nM) throughout the maturation stage revealed the minimum maturation (26.43%) and polar body rates (23.6%). Moreover, the addition of 9-cisRA at a level of 5 nM significantly increased the cytoplasmic and nuclear growth in buffalo oocytes [4]. Accumulating evidence suggests that RA might have favorable or detrimental influences during oocyte maturation and embryo development in most livestock species. Similarly, the maturation of oocytes with 0.5 µM of 9-cis RA or 0.3 µM of retinyl acetate improved the developmental capacity in goat embryos [26]. In goats, Pu et al. [1] indicated that significant improvements in the nuclear oocyte maturation and survival rates were reported after supplementing the maturation media with 10 or 100 nM of AtRA, compared to those in the control group. On the other hand, a higher level of AtRA (1000 nM) had no beneficial impacts on goat parthenogenetic blastocyst formation, embryonic division, or total cell counts [1]. Saadeldin et al. [7] studied the influences of RA addition (10, 20, and 40 µM) on the in vitro maturation (IVM) of the cumulus-oocyte complex of camel (*Camelus dromedarius*); the addition of 20 µM RA led to a significant reduction in the percentage of degenerated oocytes and a marked improvement in the first polar body extrusion and oocyte meiosis compared to those in the control and other experimental groups. The authors attributed this improvement to the marked reduction in the mRNA transcript levels of apoptosis-related genes by RA. Moreover, RA significantly reduced the expression of transforming growth factor beta (TGFβ) pathway-related transcripts. On the other hand, RA elevated the TGFβ expression in cumulus cells. The TGFβ pathway is suppressed by small molecule SB-431542. However, the addition of RA during IVM inhibited the suppressing influence of SB-431542 on the first polar body extrusion, oocyte meiosis I, and cumulus expansion in activated oocytes. Besides, it was observed that AtRA supplemented to the culture media for the in vitro oocyte maturation of mice improved the oocyte maturation and enhanced the developmental competence and fertilization rate [27]. It was also concluded that there was a positive relationship between the supplementation of AtRA to the in vitro culture of granulosa cell co-culture and meiosis resumption, and the formation of metaphase II oocytes [9,28]. 

Generally, it is accepted that oocytes had a great susceptibility to various levels of retinoid, which differs according to the culture conditions and species. Exposing porcine and bovine oocytes to higher concentrations of RA (500 nM) in Tissue Culture Medium-199 (TCM199) in vitro maturation media resulted in reduced nuclear and cytoplasmic maturation in association with cytotoxic effects [2,29]. In the converse line, it has been reported that basic maturation media enriched with potassium simplex optimization medium (KSOM), 500 nM RA, and follicle-stimulating hormone (FSH) enhanced the blastocyst development of bovine embryos [30]. The discrepancy of these findings could be attributed to the various utilized media with variable chemical ingredients, in which the reaction between the hormones (added to the maturation media) and RA had been approved to modify the mechanisms within oocytes [2]. Additionally, the species-specific nutritive supplies and time needed for in vitro nuclear development may be taken into consideration for these differences [31].

Vahedi et al. [32] indicated that using a moderate level of RA (1 µM) in the commercial TCM1 99 medium resulted in a significant increase in the bovine oocyte maturation. In water buffaloes, Cajuday et al. [5] indicated that the addition of 5 nM of RA to the in vitro maturation media could enhance the cumulus expansion rate (86%) compared to that in the control (72.02%). In addition, the improved developmental competence of oocytes and subsequent enhancement of blastocyst development were obtained following supplementation with 5 nM RA during the in vitro ripening, which could be attributed to an accomplished granular movement in the grown oocyte cytoplasm persuaded by RA, as is suggested to take place in bovine oocytes [2]. Additionally, supplementing 5 nM of 9-cis RA to in vitro maturation media provided a strong enhancement in growth rates compared to those in the control and the level was most increased after 50 nM RA application [33]. In this line, it was reported that adding 5 nM 9-cisRA to the culture media of bovine oocytes had a beneficial influence on the cytoplasmic maturation of vitrified bovine oocytes and thus enhanced the oocyte developmental competence [34]. The presence of RA can enhance oocyte maturation through several mechanisms, including antioxidant efficacy [35]; mitogen-activated protein kinase phosphatases [36]; increased midkine expression, which inhibits the rate of apoptosis [2,37]; inhibition of tumor necrosis factor-α production [7]; upregulates luteinizing hormone receptor expression, and enhanced secretion of FSH [34,38] (Table 1). 

It was hence clear that supplementation of low concentration of RA (up to 50 nM) to the in vitro maturation media enhances the maturation rates of various mammalian embryos except for porcine. Nevertheless, further studies will be required to adjust the suitable concentration for every species.

### 3.2. Effects of RA on Embryo Cleavage Rate

Supplementation with 5 nM RA (9-cisRA) to the in vitro maturation media of buffalo oocytes led to an increase in the cleavage rate by 13.5% compared to that in the control group [4]. Livingston et al. [39] stated that supplementation with 5 nM RA to bovine in vitro maturation media dramatically enhanced the embryonic growth, as assessed by the blastocyst/putative zygote rate (14.4% vs. 23.7%; *p* < 0.02). Interesting results have been obtained by Atikuzzaman et al. [6] who demonstrated that the highest cleavage rate of swine embryos was detected in the 5 nM retinoic group (84.30% versus 70.26% in the control group). However, higher concentrations of RA (500 nM) supplemented to in vitro maturation medium TCM199 in bovine [4] and swine [28] had non-significant influences on the cleavage rates. Moreover, Cajuday et al. [5] postulated that adding RA (1 or 5 nM) to the maturation media of buffalo oocytes improved the cleavage rate. Under elevated temperature (41 °C for the first 12 h and 38.5 °C for the second 12 h), RA supplementation at the 7.55 nM level to in vitro embryo production exhibited highly significant (*p* < 0.01) cleavage rates compared to controls (38.5 °C for 24 h) in bovine species [40]. Conversely, Islam et al. [41] state that RA (5 nM) supplemented to the in vitro bovine oocyte maturation medium did not influence the cleavage rates. This may be attributed to the activation of the gamete genome at the eight-cell stage in bovine. Until this stage, the maternal factors borne by the oocytes regulated the development and growth of the gamete; thus, supplementation of the medium and the culture condition do not influence the development rate up until cleavage [33,41]. Alminana et al. [29] found that the addition of 125 nM AtRA resulted in a significant elevation in the ratio of divided embryos. However, the highest levels of both metabolites (500 nM RA and 12,500 nM AtRA) were cytotoxic and prevented oocytes from reaching their nuclear maturation [29]. These variations could be attributed to various factors, such as differences in the in vitro maturation media, unusual hormone supplementation treatments, species-specific requirements, and the retinoid content in the oocytes. Accordingly, Gómez et al. [2] concluded that the neutral or deleterious effect of high levels of retinoids developed during the in vitro maturation may be attributed to the interaction between retinoids and FSH. Furthermore, it was reported that RA reduces FSH and enhanced the expression of luteinizing hormone receptors in porcine granulosa cells [38] (Table 1).

### 3.3. Effects of RA on Blastocyst Formation 

Livingston et al. [39] observed that the supplementation of 5 µM RA to the in vitro maturation medium tended (*p* < 0.07) to enhance the blastocyst development (blastocyst to putative zygote; 26.1% ± 2.2%) in comparison with control (21.9% ± 1.9%). This effect could be related to the improvements in the oocyte maturation rates due to attenuating oocyte tumor necrosis factor-α (*TNF-α*) mRNA expression [33]. Moreover, in swine, the higher blastocyst formation rate in vitro was reported in the 5 nM RA group compared to that in the control group [6]. Furthermore, supplementation with RA (5 nM) significantly improved the buffalo blastocyst formation rate by 38.88% compared to that in the control [5], which could be attributed to the possible activation of the RA-inducible MAP kinase phosphatase gene, polyadenylation of mRNA in oocytes, regulation of redox signaling pathways, and gene expression of midkine [5]. In addition, Islam et al. [41] detected a significantly enhanced bovine blastocyst development rate, total blastomere number, percentage of apoptotic blastomere per blastocyst, and apoptotic blastomere count per blastocyst with the addition of RA. The RA enhances the in vitro maturation of oocytes by affecting luteinizing hormone or FSH receptor transcription [38], increasing the mRNA quality and processing [42], growth factor signalling [2] by endogenous oxidative-stress conservation mechanisms [43], and/or the prevention of apoptosis [33].

Alminana et al. [29] reported that a higher concentration of RA (500 nM) significantly reduced the maturation rates of porcine oocytes raised in vitro. The authors attributed the positive effects of RA to the upregulation of luteinizing hormone receptor expression and the enhancement of FSH secretion, which could be related to more complete granular migration in the matured oocyte cytoplasm induced by RA. This migration provides a block to polyspermy once migrated cortical granules (CGs) have been released. AtRA administration to superovulated ewes enhanced the embryonic viability and positively influenced embryonic development [15]. Moreover, Hidalgo et al. [44] indicated that the in vitro maturation of bovine oocytes in the presence of 9-cisRA (5 nM) resulted in higher oocyte competence, blastocyst formation and hatching rate, pregnancy rate, and cell count and proportion of cells in the inner mass of day seven blastocysts. Moreover, the supplementation of 7.55 nM RA to bovine oocytes under elevated temperature (41 °C) significantly improved the embryonic stages (2–4 cells, 8–16 cells, morula, and blastocyst) compared to those in the control group [40]. This enhancement of embryonic stages under thermal stress reflects the potential function of RA forms for overcoming the deleterious effects of elevated temperature. On the other hand, a higher level of AtRA (1000 nM) had no positive effect on goat blastocyst formation [1]. However, Lima et al. [30] mentioned that 500 nM 9-cisRA supplemented to bovine maturation medium elevated the blastocyst formation, but the findings obtained in pigs [6] and buffaloes [4] showed that 500 or 200 nM 9-cisRA was actually slightly toxic to oocytes. Collectively, it could be concluded that RA supplementation to in vitro maturation media significantly increased blastocyst formation under some conditions (Table 1).

## 4. Different Modes of Action of RA and Retinol

### 4.1. Impact of RA on Mitochondrial Membrane Potential Activity and ROS Level 

The mitochondrial membrane potential of oocytes is considered to be a remarkable indicator of mitochondrial function. The enhancement of mitochondrial membrane potential activity was reported with the in vitro supplementation of 5 nM RA to the maturation media of buffalo oocytes. However, buffalo oocytes matured with 50 or 200 nM RA showed reduced mitochondrial activity compared to that in the control or 5 nM RA groups [4].

The growing capacity of the oocyte, as well as the preimplantation embryonic development, was greatly associated with the potency and arrangement of mitochondria [45]. The mitochondria is responsible for using oxidizable substrates to produce ATP, which drives all cellular events, including metabolic, translation and transcription events; these events are pivotal for normal cytoplasmic and nuclear maturation [46]. Although mitochondria are considered an energy source via the oxidative phosphorylation path, it also is the principal source of ROS. High concentrations of ROS caused cell apoptosis via disturbance of the mitochondrial role in oocytes and embryos [47]. Internally generated or externally supplemented antioxidants have essential roles in sustaining the antioxidant/oxidant equilibrium of oocytes in the in vitro culture medium. 

Many antioxidants, including enzymatic and non-enzymatic, were tested within in vitro cultured oocytes and embryos in various mammalian species, such as sheep, ewes, and rats and represented an effective agent against ROS [48]. Gad et al. [4] showed that the administration of 5 nM RA resulted in lower ROS levels in the maturation media. However, a higher level of RA led to electron outflow from the mitochondrial membranes and consequently diminished the mitochondrial function, resulting in augmented ROS generation [49]. Enriched bovine oocyte maturation media with different isomers of RA caused a significant decrease in the level of ROS [50]. Therefore, the protective effects of RA have been explained by its capability to clear ROS in in vitro cultured oocytes [4]. Furthermore, retinoids can maintain suitable endogenous antioxidant enzyme levels, which can be utilized to defend oocyte maturation and embryo development by resisting the deleterious effects of ROS generation [43]. Pu et al. [1] found that adding AtRA (10 or 100 nM) to in vitro goat oocyte maturaton media led to a decreased ROS generation; thereby, AtRA has a positive impact as an antioxidant agent on goat oocyte nuclear growth, beside the reduction in apoptotic cumulus cell levels.

### 4.2. Impact of RA on Apoptosis

Cumulus cells are a type of granulosa cell that surrounds the oocyte and plays a vital part in the cytogenesis and maturation of the oocyte via providing nutrients and regulatory signals [6]. Cumulus cells in cumulus oocyte-complexes are subjected to apoptosis during in vitro oocyte maturation [51]. It has been reported that the development of oocytes may induce cumulus cell apoptosis [52]. In swine, Atikuzzaman et al. [6] noticed that 5 nM 9-cisRA induced cumulus cell expansion by suppressing their up-expression of prostaglandin-endoperoxide synthase 2. Pu et al. [1] clarified the potentially favourable impact of RA on cumulus cells and goat oocyte via the inhibition of cumulus cell apoptosis; compared to in the control group, RA significantly reduced apoptosis in goat cumulus cells. This anti-apoptotic effect might be related to the upregulation of the anti-apoptotic protein B-cell lymphoma 2 (BCL-2) (1.5-fold) and downregulation of the pro-apoptosis gene *caspase-8* after the treatment of cumulus cells with RA [1]. Moreover, Saadeldin, et al. [7] reported a similar antiapoptotic effect of RA on the cumulus oocyte-complex in camel. ROS can induce lipid peroxidation and enzyme inactivation, which would cause cell damage by promoting hydroxyl radical outcomes [53]. AtRA diminished the ROS levels in oocytes during in vitro maturation; therefore, AtRA enhanced the growth rates of the oocytes. The detected improvements in oocyte maturation could be attributed to the decrease in ROS concentration via the upregulated transcription of catalase in cumulus cells in the media, where ROS was among the abundant factors affecting oocyte competence [1]. Moreover, AtRA minimized goat cumulus cell apoptosis by defending cumulus cells from ROS damage [1]. Moreover, RA induced efficient protection against the in vivo cardiotoxic effect of the chemotherapeutic agent doxorubicin through its antioxidant and anti-apoptotic signalling [54]. In view of the above findings, it could be suggested that RA decreased cumulus cell apoptosis during the in vitro maturation of different species. 

## 5. Effects of RA on Antioxidant-Related Genes

**Figure 3:**
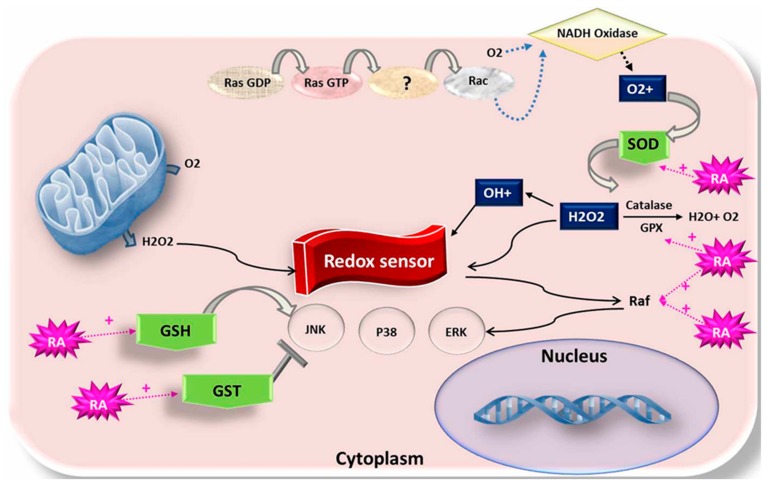
Schematic representation of the redox pathway and the mechanism of action of retinoids in the cytoplasm during embryogenesis.

The role of RA as a potential antioxidant agent (Figure 3) is thought to be due to its influence on the gene level; RA is able to modulate the expression of many nuclear genes via the homo or hetero-expression of the RXR and RAR receptors next to their binding with RA [55]. During in vitro maturation, oocytes collected by aspiration from elderly animals (obtained from the slaughterhouse) suffered from mitochondrial dysfunction, reduced ATP generation, increased ROS production, and a higher expression of apoptosis-related genes or downregulated expression of antioxidant genes [56]. This alteration at the transcriptome gene level could be reversed by using mitochondrial nutrients. Thus, anti-apoptotic as well as oocyte-linked genes were proven to be up-modulated in in vitro oocytes grown with RA [2]. Further, it has been stated that RA enhances polyadenylation action [42]. Gad et al. [4] concluded that the addition of 5 or 50 nM RA to the maturation media resulted in the upregulation of antioxidant-modified genes including glutathione Peroxidase 4 *GPX4),* catalase ( *CAT*)*,* heme Oxygenase 1(*HOMX1*)*,* peroxiredoxin-1 (*PRDX1*)*,* and superoxide dismutase-1 (*SOD1*) in matured buffaloes oocytes. However, a higher level of RA supplementation caused an obvious down-modulation of antioxidant-candidate genes, except for peroxiredoxin (PRDX1) [4]. During the development of the oocyte from the meiotic I (MI) to meiotic II (MII) divisions, its expression outcomes gradually declined and became highly conditional on the reserved maternal proteins and RNA [37]. Considering this, several approaches have been suggested to enhance oocyte development and competence. 

SOD1 is considered a valuable gene that boosts oocytes resisting oxidative stress as it has the capability to participate in the deterioration of ROS, which are generated through adverse circumstances. Thus, RA was approved to guard breakdown in protein and transcript values of SOD in cultured buffalo and rat oocytes [57,58]. Such overprotection reinforced the endogenic antioxidant guarding mechanisms and later on minimized ROS values. Results of multiple studies indicate that the expression of SOD1 has been increased, which elucidated that the inclusion of antioxidant or RA to oocyte maturated media leads to up-modify such genetic materials as antioxidant attitude [4,59,60]. Additionally, SOD1 was important for proceeding step that protected ovarian cells from free radicals such as hydrogen peroxide (H_2_O_2_) and oxygen radicals (O.) then scavenged by catalase or GPX [43]. 

Glutathione peroxidase 4 is an intracellular antioxidant enzyme that directly decreases peroxidized phospholipids even if they are incorporated in lipoproteins and biomembranes [61,62]. It has been reported that this enzyme could play a pivotal role in regular murine embryogenesis [62,63] and apoptosis regulation [64]. Moreover, only *GPX4* mRNA expression in sperm cells was statistically significantly lower when asymmetric embryos were observed [65]. Gad et al. [4] found that the addition of 5 or 50 nM RA to in vitro maturation media significantly upregulated *GPX4* gene of matured buffalo oocytes in relation to 200 nM 9-cisRA and control groups. 

Peroxiredoxin1 (Prdx1) was recognized as an antioxidant enzyme which affiliating to the peroxiredoxin proteins and regulated cellular ROS production by catalyzing the reduction of alkyl hydroperoxide and H_2_O_2_ [66]. The decrease expression of Prdx1 in maturated oocyte as affected by retinoic supplementation supported antioxidant capacity [4,67], played as a down-modulator of the RA impression throughout embryogenesis [66]. The study that has been made by Jeong et al. [68] revealed that PRDX1 is fateful to regulating lipophagic flux and maintaining macrophage cholesterol homeostasis against oxidative stress. 

Formed heme-oxygenase 1 (HMOX1) were essential antioxidant enzymes which played the main physiological role in the cellular cell defence. They were associated in oxidative biodegradation of heme into equal molar quantities of carbon monoxide, biliverdin, and ferrous irons, which principally after conversion into bilirubin was recognized to have powerful antioxidant impacts [69]. The transcription of HMOX1 gene was modified by the Keap1-Nrf2 mechanism later attachment of Nrf2 to concurrence attachment sequence and initiated an overflow of incidents, which finally gave strong over-shielding in the face of oxidative changes [70]. The significant influence of HMOX1 through oocyte growing has been clarified when female Hmox1/mice exhibited minimum percentages of oocyte growing and generally decreased fertility in relation to the wild-type mice [71]. Moreover, Gad et al. [4] showed that buffalo oocytes maturated with 50 and 200 RA exhibited higher significant of the expression of HMOX1 gene.

## 6. Effects of Retinoic Acid on Prostaglandin-Endoperoxide Synthase (PTGS)

The two forms of prostaglandin-endoperoxide synthase (PTGS1 and PTGS2) vary in their features of transcription and thereby their modification in mammalian cells [73]. During normal physiological status, the PTGS1 mRNA and protein were established in many tissues to enclose the synthesis of prostaglandins for so-called housekeeping functions [6]. PTGS1 was significantly decreased (5.86-fold less) in the RA (5 nM) group and increased 8.38-fold in the control group [6]. PTGS2 is considered a key factor affecting the cumulus cell expansion, and consequently enhances oocytes competence and embryo development in porcine [6]. It has been described that the higher expression of PTGS2 was found in pre-ovulatory follicles (mural granulosa cell origin) in many species for instance; pigs, cattle and horse. Also, PTGS1 and PTGS2 have been established in mature and immature cumulus granulosa cells in the porcine cumulus oocytes complex [6]. Expression of PTGS2 in the cumulus-oocyte complex has been related to the level of prostaglandin in the maturation medium [74]. It was reported that prostaglandin biosynthesized by PTGS2 has a pivotal role in ovulation, built on the proof that minimizing of PTGS2 throughout the pre-ovulatory time, depressed ovulation in bovine [75], as well as in several other mammalian species [6]. It has been found that RA with a level of 5 nM during in vitro maturation can steady the transcription of prostaglandin-endoperoxide synthase2 (PTGS2). This influence reflects the potential role of RA with 5 or and 50 nM 9-cisRA against transcripts degradation [4]. In contrast, Atikuzzaman et al. [6] observed that 5 nM RA can restrain PTGS2 transcription in cumulus cells in comparison to the control group. This level of RA (5 nM) supplemented to in vitro maturation media stabilized the expression of PTGS2 and diminished the excessive amount of prostaglandins synthesis. Consequently, in vitro oocyte maturation and following porcine embryonic growth were enhanced by supplementation of 5 nM RA [6].

## 7. Conclusion and Future Perspective

Retinoids are a natural source with abundant potential for use in assisted reproductive technology. RA had many precious antioxidant activities for enhancing the oocyte competence and embryogenesis in different animal species. Its therapeutic and biological activities have been established, but embryologists just have begun to uncover many health benefits during the oocyte and embryo development. On the other hand, at the level of cellular process, retinoids were not sufficiently studied. Further research either in vitro or in vivo and for clinical applications is needed to pave the way for RA supplementation in assisted reproductive technology. 

## Figures and Tables

**Table 1:** Retinoic Acid (RA) supplementation in IVM and the effect on oocyte maturation, fertilization/cleavage, and blastocysts development.

Items	Species	Effective Dose	Mechanism of Action	References
1. Oocyte maturation	Water buffalo	5 nM (9-cisRA)	Enhancement in mitochondrial membrane potential activity	[4]
	Goat	10 or 100 nM (All-*trans* RA)	B-cell lymphoma 2 (BCL2) upregulation and Caspase-8 was downregulated	[1]
	Camel	20 µM (RA)	Reduction of the mRNA transcript levels of apoptosis-related genes Reduction of the expression of transforming growth factor beta (TGFβ) pathway-related transcripts	[7]
	Mouse	2 and 4 µM (All-*trans* RA)	Modulatory effects on the gene expression of gonadotropin receptors, midkine (Mk), cyclooxygenase-2 and nitric oxide syntheses in cumulus-granulosa cells	[27]
	Mouse	2 µM (RA)	RA may stimulate follicle-stimulating hormone (FSH) for induction of luteinizing hormone (LH) receptors RA regulates progesterone generation and reduces adenosine 3′,5′cyclic monophosphate (cAMP) levels. It could also protect oocyte against oxidative stress induced by apoptosis through reduction of free oxygen radicals and interaction with other antioxidant compounds.	[9]
	Mouse	2 µM (All-*trans* RA)	Cortical granule migration as a maturation clue could be affected by RA since RA improves granular migration.	[28]
	Bovine	5 nM 9-cis RA	Attenuating oocyte tumor necrosis factor-α (TNF-α) mRNA expression.	[33]
	Porcine	5 and 50 nM RA 125 and 1250 nM ROH	Upregulates luteinizing hormone receptor expression and enhance the secretion of follicle stimulating hormone. And could be related to more complete granular migration in the matured oocyte cytoplasm induced by RA. This migration provides a block to polyspermy once migrated cortical granules (CGs) have been released.	[29]
	Canine	5 nM 9-cisRA	The relative expression of Bcl-2 and Bax in the oocytes seemed to be associated with different developmental competence.	[37]
	Porcine	5 nM 9-cisRA	RA reduces FSH and created the expression of luteinizing hormone receptor in porcine granulosa cells	[6]
	Bovine	1 μM (All-trans RA)	Increase in midkine (MK) mRNA. It is also found that MK suppressed apoptosis in the cumulus cells during the IVM period of bovine COCs. Although we did not assay the effect of RA on MK expression, it is possible that RA enhances oocyte nuclear maturation through the production of MK in cumulus cells in the in vivo and/or in vitro maturation of COCs.	[32]
	Water buffalo	5 µM RA	The possible activation of RA-inducible mitogen-activated protein kinase (MAPK) gene, polyadenylation of m-RNA content in oocytes, regulation of redox signaling pathways and gene expression of midkine.	[5]
	Bovine	5 nM 9-cisRA 500 nM RA in TCM199 is toxic	Could be related to more complete granular migration in the matured oocyte cytoplasm induced by RA	[2]
	Bovine	500 nM RA in potassium simplex optimization medium (KSOM)	Can improve nuclear and cytoplasmic competence, consequently enhancing the developmental competence of the oocyte to achieve a later stage of embryonic development, a result obtained in the present study. The addition of RT does not modify bovine oocyte nuclear maturation kinetics and can improve the effect of growth factors; furthermore, supplementation of maturation medium with RA has a beneficial effect on cytoplasmic maturation.	[30]
	Bovine	5 nM of 9-cisRA	Enhances the developmental competence and cortical granules distribution of meiosis vitrified bovine oocyte. Therefore, adding RA in IVM medium can decrease the ultrastructural changes during vitrification and can improve the efficiency of bovine oocyte vitrification.	[34]
	Bovine	7.5 µM retinol	Expansion of cumulus cells and an increase in MII nuclear maturation. Several transcripts of retinoid receptors have been identified in bovine oocytes and embryos from the 2-cell to the hatched blastocyst stage thought to be targets of the transcriptional influence of retinol.	[40]
2. Embryo cleavage rate	Water buffalo	5 or 50 nM (9-cisRA)	Could be attributed to the enhanced oocyte maturation rates	[4]
	Bovine	5 nM retinol	Could be related to the improvements in the oocyte maturation rates	[39]
	Porcine	5 nM 9-cisRA	RA reduces FSH and created the expression of luteinizing hormone receptor in porcine granulosa cells	[6]
	Bovine	7.5 µM retinol	Could be related to the improvements in the oocyte maturation rates	[40]
	Water buffalo	(1or 5 nM) RA	Could be attributed to the enhanced oocyte maturation rates	[5]
	Bovine	5 nM RA	No Effect	[41]
	Porcine	125 nM all-*trans* retinol	Could be related to the improvements in the oocyte maturation rates	[29]
3. Blastocyst formation	Bovine	5 µM retinol	Could be attributed to the enhanced oocyte maturation rates	[39]
	Porcine	5 nM 9-cisRA	Could be related to the improved oocyte maturation rates	[6]
	Bovine	5 nM 9-cisRA	Could be related to the improvements in the oocyte maturation rates	[41]
	Porcine	1 µM retinoid	Retinol reduces FSH and created the expression of luteinizing hormone receptor in porcine granulosa cells	[38]
	Bovine	7.5 µM retinol	Increasing mRNA quality and processing	[40]
	Bovine	5 nM 9-cisRA 500 nM RA in TCM199 is toxic	Could be related to the improved oocyte maturation rates and growth factors signaling	[2]
	Porcine	5 nM RA	Protection from oxidative damage, which is a major cause of in vitro embryonic wastage	[29]
	Bovine	500 nM 9-cisRA in potassium simplex optimization medium (KSOM)	Could be attributed to the enhanced oocyte maturation rates	[30]
	Water buffalo	5 µM RA	RA act on cells at the transcriptional level; hence, it can modify transcription activity in the COC to influence cytoplasmic maturation and the subsequent capacity of the oocyte to progress in development, including cleavage.	[5]
	Bovine	0.7 μM (All-trans RA)	RA triggered an increase in the apoptotic frequency of the inner cell mass. RA reduced the necrotic index. Na/K-ATPase α1-subunit mRNA concentrations (analyzed by real-time PCR) increased after hatching and showed dependence on retinoid activity.	[72]
	Bovine	5 nM (9-cisRA)	Could be attributed to the enhanced oocyte maturation rates	[44]
	Goat	1000 nM (All-*trans* RA)	No good effect	[1]
